# Toward a Psychology of Social Change: A Typology of Social Change

**DOI:** 10.3389/fpsyg.2017.00397

**Published:** 2017-03-28

**Authors:** Roxane de la Sablonnière

**Affiliations:** Social Change and Identity Laboratory, Department of Psychology, Université de MontréalMontréal, QC, Canada

**Keywords:** identity, inertia, normative structure, social change, social structure, stability, pace of change, psychology of social change

## Abstract

Millions of people worldwide are affected by dramatic social change (DSC). While sociological theory aims to understand its precipitants, the psychological consequences remain poorly understood. A large-scale literature review pointed to the desperate need for a typology of social change that might guide theory and research toward a better understanding of the psychology of social change. Over 5,000 abstracts from peer-reviewed articles were assessed from sociological and psychological publications. Based on stringent inclusion criteria, a final 325 articles were used to construct a novel, multi-level typology designed to conceptualize and categorize social change in terms of its psychological threat to psychological well-being. The typology of social change includes four social contexts: Stability, Inertia, Incremental Social Change and, finally, DSC. Four characteristics of DSC were further identified: the pace of social change, rupture to the social structure, rupture to the normative structure, and the level of threat to one's cultural identity. A theoretical model that links the characteristics of social change together and with the social contexts is also suggested. The typology of social change as well as our theoretical proposition may serve as a foundation for future investigations and increase our understanding of the psychologically adaptive mechanisms used in the wake of DSC.

“Change—*extremely rapid social change*—is the most important fact of life today”

(Nolan and Lenski, 2011, p. xiii).

Zoia is a lively 75-year-old *Baboushka*. Her eventful life has seen her experience some less-than-welcome adventures, but she has always managed to adapt to unfamiliar circumstances. After completing her studies in Moscow, she was, like many other young educated Russians, deported by USSR authorities to another state. Her destination was Frunze (later renamed Bishkek), a land in Central Asia warmer than hers and made slightly cooler by its unfamiliarity. Despite the diversity of Frunze, with ethnic Kyrgyz, Ukrainians, and other Slavic groups forming sizeable minorities, the Russian population remained a majority. During the Soviet era, Zoia was told that she lived in one of the most powerful countries in the world, where crime rates were low and the population enjoyed decent education and food supply, as well as the opportunity to save money for retirement.

The diversity of ethnicities eventually bred great tension, and the collapse of the Soviet Union in the early 1990s deeply affected Zoia's life. At the age of 54, she learned that her country was in ruins, that her rights as a Russian were diminished and that her language was widely frowned upon within the newly formed Kyrgyz Republic, Kyrgyzstan. Meanwhile, the disorganized authority allowed for an explosion in crime rates and increasing scarcity of resources. Zoia lost all of her life savings. The money she earned was no longer sufficient to cover basic necessities. Despite her position as a chief engineer, Zoia was forced to work a second job selling newspapers at the corner of her street just to make ends meet.

Although Zoia's story may seem uniquely dramatic, it is only one among over one billion (Sun and Ryder, 2016). Social change is indiscriminately pervasive and global—restricted to neither developing nor western worlds (e.g., Ponsioen, 1962; Smith, 1973; Chirot and Merton, 1986; Zuck, 1997; Sztompka, 1998; Fukuyama, 1999; Weinstein, 2010; Nolan and Lenski, 2011; Greenfield, 2016). Dramatic social change (DSC) is the new normal and can be witnessed presently across a multitude of contexts from political and economic upheaval, to desperate mass migration, and from natural or human disasters to technological advances.

Social change has always been a field of great interest for the social sciences, especially among sociologists since it seems that “all sociology is about change” (Sztompka, 1993, p.xiii; see also Sztompka, 2004). Many sociology texts have entire sections devoted to social change (e.g., Bauman, 2003; Latour, 2005; Hewitt et al., 2008; Giddens et al., 2011) all aimed at addressing one main question: *What leads to social change*? Many sociological theories have been suggested to explain the different “macro” processes associated with the onset of revolutions, social movements, or important technological changes. A “macro” theory focuses on the structural factors or defining events that contribute to DSC and are useful when considering how social changes are brought upon an entire group, community, institution, nation, or indeed society as a whole. The macro approach, however, is seriously limited when it comes to “micro” processes, which focus on the equally important question of the consequences of social change, or, in other words, how individual group members are impacted by social change (e.g., Rogers, 2003). Thus, the exclusive research focus on macro processes has left unanswered the pivotal question: *What are the psychological consequences of social change?*

Given the potentially dire consequences of DSC, it is surprising that psychologists have neglected it as a topic of rigorous academic pursuit, particularly given the current reality of vast globalization and massive immigration. To date, research focusing on the impact of social change on the well-being of individuals has not been clearly established (Kim, 2008; Liu et al., 2014). Moreover, the adaptation mechanisms that people develop when coping with such contexts remain largely unknown (Pinquart and Silbereisen, 2004).

The goal of the present paper is to argue that psychology needs to focus on the *psychology of social change* (de la Sablonnière et al., 2013; de la Sablonnière and Usborne, 2014). I argue that the bridge between the “macro” processes of social change and the “micro” processes of its psychological impacts have yet to be built. I suggest that social scientists must first focus on conceptualizing social change in a manner that includes both macro and micro processes in order to understand individuals' adaptation to social change. Thus, as the first *step* in moving toward a psychology of social change, I target what is considered the most difficult challenge: conceptualizing social change.

First and foremost, conceptualizing social change requires untangling the complexity of the topic by formulating a *typology of social change* (see Table 1). To that end, a large-scale meta-review that assembled original perspectives, theories and definitions of social change within both the sociological and psychological literature was performed. The typology of social change that emerged distinguishes four separate social contexts associated with social change: stability, inertia, incremental social change, and DSC. DSC, because of its frequency in today's world, and because it is threatening to people, requires special attention. Thus, the proposed typology of social change drills deeper and articulates four necessary characteristics for a change or an event to be labeled as “dramatic social change”: rapid pace of change, rupture in social structure, rupture in normative structure, and threat to cultural identity. Finally, I come full circle by proposing a theoretical model that links together the four characteristics of DSC within the proposed typology of social change (see Figure 1). In sum, the typology of social change I am suggesting can be useful to create a theoretical consensus among researchers about what social change is that perhaps will allow for a coordinated, evidence based strategy to address the psychology of social change.

**Table 1:** **The typology of social change**.

**Social contexts**	**Definition**
Stability	A situation where an event, regardless of its pace, does not affect the equilibrium of a society's social and normative structures nor the cultural identity of group members. The event, may, however, impact an isolated number of individuals.
Inertia	A situation where an event, regardless of its pace, does not either reinstate the equilibrium of a society's social and normative structures or clarify the cultural identity of group members.
Incremental social change	A situation where a slow event leads to a gradual but profound societal transformation and slowly changes the social and/or the normative structure or changes/threatens the cultural identity of group members.
Dramatic social change	A situation where a rapid event leads to a profound societal transformation and produces a rupture in the equilibrium of the social and normative structures and changes/threatens the cultural identity of group members.

**Figure 1:**
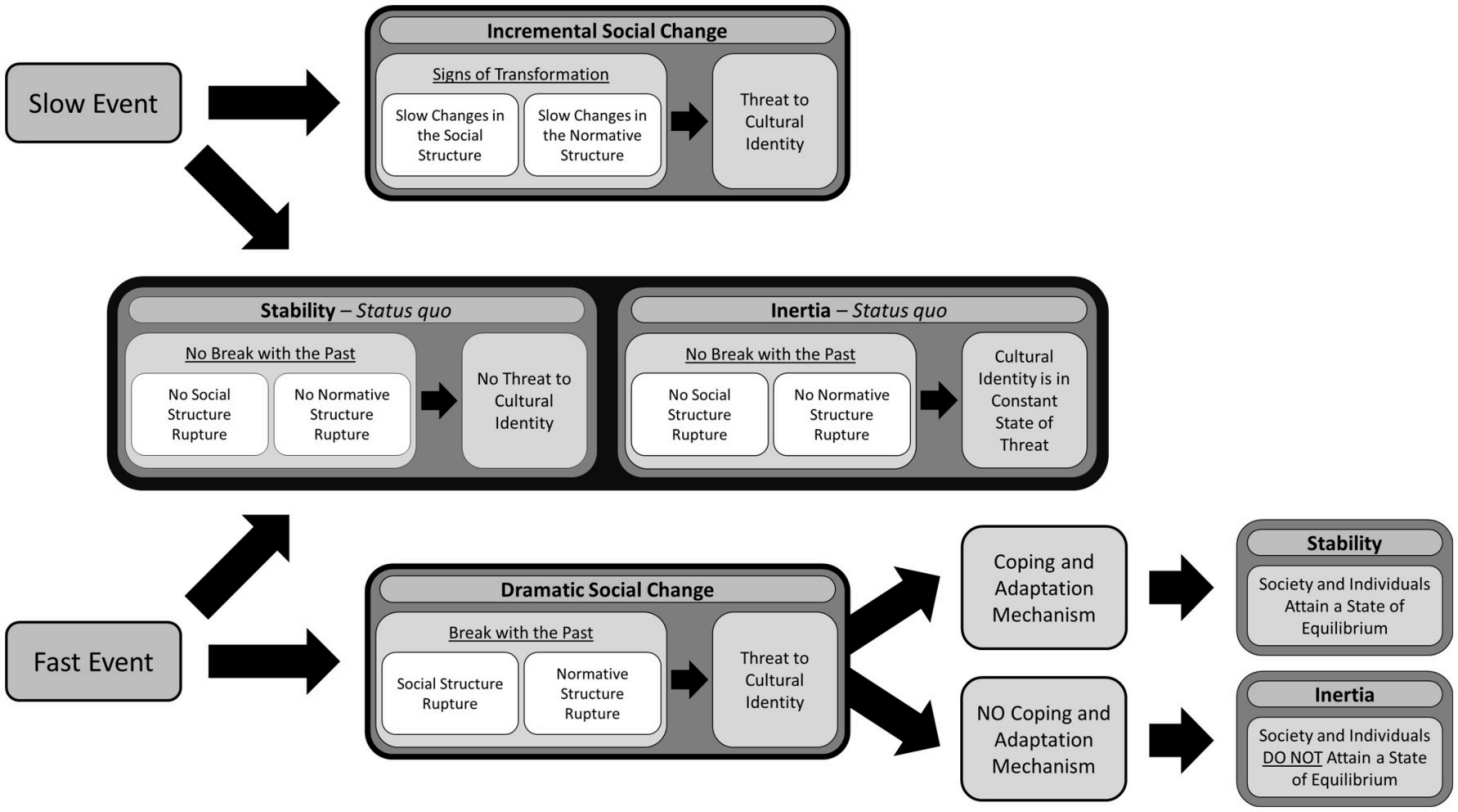
**Proposed theoretical model**.

## Social change in sociology and psychology

Today, the field of sociology is at the forefront of social change theory and research, with a particular focus upon the factors that constitute and are prerequisites to social change. Within the sociological literature, three main theories have been championed for their attempt to explain social change: *Evolutionary Theory, Conflict Theory*, and *Functionalist Theory*. Each theory is characterized by key descriptive interpretations in Table 2 where a global overview of the conceptualization of social change is offered^1^.

**Table 2:** **Theories of social change in sociology**.

**Theories**	**Perspective on social change**	**Key authors**
Evolutionary theory	Society moves in a linear direction from a simple to a more complex structure.	Comte, 1853/1929; Spencer, 1898; Pareto, 1901/1968
Conflict theory	Individuals and their groups fight to maximize their benefits. Society is in a constant state of disequilibrium.	Marx and Engels, 1848
Functionalist theory	Society is in a constant state of equilibrium. When a change occurs in one part of society, adjustments are made. Social change occurs when the equilibrium is compromised due to the rapidity with which events occur.	Durkheim, 1893/1967; Parson, 1951

Despite the first appearance of “social change” in the psychological literature more than 70 years ago, only a few isolated psychologists have focused on social change *per se* and even fewer have offered a clear definition or conceptualization of the concept. The first paper that defined social change was published in the *Academy of Political and Social Science* and was entitled *Psychology of Social Change*. Social change was defined as “always a slow and gradual process” (Marquis, 1947, p. 75). From that point in time to the dissolution of the Soviet Union in 1991, there have been very few attempts to reintroduce social change into the field of psychology (e.g., Pizer and Travers, 1975; Schneiderman, 1988). However, after the dissolution of the Soviet Union and the fall of the Berlin Wall, there has been a small surge of research on social change in psychology. For example, several edited books (e.g., Thomas and Veno, 1992; Breakwell and Lyons, 1996; Crockett and Silbereisen, 2000) and special issues of journals (Silbereisen and Tomasik, 2010; Blackwood et al., 2013) have focused exclusively on social change and on people's reactions to it. For clarity purposes, Table 3 attempts to summarize the various theories or perspectives in different subfields of cultural and social psychology while Table 4 attempts to do so in subfields of psychology.

**Table 3:** **Theories and perspectives addressing social change in social psychology**.

**Theory**	**Perspective on social change**	**Key authors**
Social Identity Theory (SIT)	Social identity relies on two aspects that may be associated with social change. First, SIT is a theory of social structure that is based on perceptions of legitimacy, stability, and permeability. Second, SIT proposes identity management strategies such as collective action whereby minority groups aim to maintain or acquire a positive and distinctive social identity.	Tajfel and Turner, 1986
Social Dominance Orientation (SDO)	In terms of SDO, social change can be interpreted as the opposition of hierarchy-enhancing attitudes in individuals with high SDO and hierarchy-attenuating ones in individuals with low SDO.	Sidanius and Pratto, 1999
Relative Deprivation Theory (RDT)	RDT can be applied to social change in two distinct ways. First, collective relative deprivation occurs when people compare their group to other groups and feel that their group is worse off which will motivate them to improve their status by means of collective action. Second, in times of DSC, people are usually confronted with a unique situation that results in confusion and the loss of social cues. It is therefore easier and more relevant for them to compare their group's present situation to their group's status at another well-defined time period, than to compare their group with another group. Recent research proposes the use of a historical trajectory when assessing one's group's collective relative deprivation.	Runciman, 1966; de la Sablonnière et al., 2009a, 2010
Immigration and Identity Integration (III)	Immigration is a form of social change that requires human adaptation. Research in this field has demonstrated that individuals who simultaneously identify with their culture of origin and with the receiving group's culture and also desire contact with both cultures experience the highest levels of well-being.	Benet-Martínez and Haritatos, 2005; Berry, 2005; Amiot et al., 2007
Identity Process Theory (IPT)	IPT explores the structure of an individual's identity and the coping strategies used when facing an identity threat or change that results from social change.	Breakwell, 1986
System Justification Theory (SJT)	SJT is a theory that explains how to preserve the status quo. It's more a theory of stability than of social change. Both advantaged and disadvantaged individuals endorse system-justifying ideologies, to preserve the existing social structure.	Jost et al., 2004
Identity Threat Theory (ITT)	In ITT, when a threat to identity occurs as a result of social change, individuals will regulate the structure of their identity by restoring the imbalance and modifying their identity through different processes that include integrating the new elements into their identity and assigning a positive or negative valence to them.	Steele et al., 2002
Adjustment to Change Theory (ACT)	ACT considers how individuals adjust to social change and argues that factors such as social support and the nature of the event predict the way individuals and groups evaluate social change.	Goodwin, 2006

**Table 4:** **Theories addressing social change in subfields of psychology**.

**Theory**	**Perspective of social change**	**Key authors**
Cultural and evolutionary psychology	Focuses on how social change and human biology are linked and aims to identify how social change influences human genetics and the way humans adapt to these changes.	Feldman and Laland, 1996; Laland et al., 2000
Developmental psychology	Research in this field has demonstrated that social change has the potential to impact developmental stages for children and adolescents as well as their identities and well-being.	Pinquart and Silbereisen, 2004; Greenfield, 2009, 2016
Industrial/organizational psychology	Focuses on organizational change as a form of social change. Three main themes emerge from this field: how to successfully implement organizational change, how to limit the negative impact of organizational change and understand the psychological processes of people who are confronting organizational change.	Kanter, 1991; Burke and Litwin, 1992; Sanzgiri and Gottlieb, 1992; Meyer and Allen, 1997; Reichers et al., 1997

## Limitations of current research and conceptualization of social change in sociology and psychology

As indicated in the summary tables, both contemporary and traditional theorists in sociology and psychology have addressed social change through a variety of macro sociological or societal lenses, and equally from a plethora of micro, psychological, or individual perspectives. Theory and research thus far has demonstrated that social change is a complex entity (e.g., McGrath, 1983; Buchanan et al., 2005; Subašić et al., 2012) that can be conceptualized in many diverging (and confusing) ways. The challenge associated with defining social change may well be to explain why it is an understudied phenomenon (de la Sablonnière et al., 2013) and highlight the challenge of moving forward in studying its psychological impact on ordinary people. The typology of social change presented here offers an initial attempt at clarifying the meaning of social change from a psychological perspective. That is, I focus on an individualistic perspective, but attempt to address the role that macro processes play in terms of our more micro or psychological focus. Here, I discuss three main issues that point to the necessity to properly conceptualize DSC.

First, and most importantly, the conceptualization and understanding of social change does not reach a consensus within the scientific literature (e.g., Coughlin and Khinduka, 1976). Furthermore, few scientists define precisely what they mean when using the concept (e.g., Saran, 1963). For example, when social change is studied from a social identity theory perspective (Tajfel and Turner, 1986), or a sociological conflict theory perspective, social change is conceptualized almost exclusively in the context of collective action (Krznaric, 2007). In light of this, collective action is defined as a means for group members to achieve an improved social position for their group in the social hierarchy (Taylor and McKirnan, 1984; Batel and Castro, 2015; de Lemus and Stroebe, 2015). In contrast, cultural psychology and developmental psychology conceptualize social change in a broader manner (e.g., societal transformations such as the fall of the Soviet Union; immigration) where change is not limited to the context of intergroup conflict (Pinquart and Silbereisen, 2004; Sun and Ryder, 2016). The fact that there is divergence in conceptualizing social change is preventing coordinated research on social change, because not all types of social change are considered. With some theories (e.g., relative deprivation theory, social identity theory, evolutionary theory, conflict theory), social change is conceived mostly as an autonomously controlled and unidirectional process toward group change; these conceptualizations do not account for social changes that are outside of human control, such as natural disasters (e.g., Coughlin and Khinduka, 1976). Equating social change with collective action (see Stroebe et al., 2015), for example, neglects uncontrollable social transformations such as socio-political reforms and natural disasters over which individuals or groups exert no control. Indeed, the majority of individuals who experience DSC have little control over such events. Since previous classifications can only explain some instances of social change, a theory that would clarify the characteristics required in conceptualizing DSC for all types of change has become a necessity.

The second issue that points to the need for a typology of social change is that not all social contexts associated with social change (i.e., stability and inertia) were considered in previous scientific literature. Most theoretical and empirical work on social change in both sociology and psychology has focused on either incremental social change or DSC (e.g., Andersson et al., 2014; Bernstrøm and Kjekshus, 2015). However, in order to have a complete theory or typology of social change, it is also necessary to take into account social contexts where there is no social change, contexts of either *stability* or *inertia* (Table 1). Knowing about incremental social change, inertia and stability, as well as how they relate to DSC is psychologically critical. A clear definition of the four social contexts of social change can facilitate finding solutions for the population to not only the consequences associated with DSC, but also the considerable and potentially unique challenges associated with each of these social contexts (see Abrams and Vasiljevic, 2014). For example, a society in a state of inertia may be misconceived as a society in a state of DSC if no clear understanding of each social context is achieved. In inertia, there might be less hope for reverting to a healthy society and consequently less long-term goals that are developed, whereas a time of DSC, such as a political revolution, may provide some hope for the future and some possibilities for some concrete long-term goals. Although the main focus of our paper is DSC, the full spectrum of social contexts associated with social change is presented. A more comprehensive theory of social change capable of accounting for stability, inertia as well as incremental and DSC is required to fully understand the psychological processes and ramifications of social change. Moreover, it is important to define stability, inertia, and incremental social change because they serve as a base for comparison or contrast to DSC. As Calhoun notes: “To understand social change, thus, it is necessary also to understand what produces social continuity” (Calhoun, 2000, p. 2642).

Finally, the third issue that pushes me to develop a typology of social change is that, mainly in sociology, a specific event that can be characterized as social change can be interpreted in light of different *theories* of social change. Let us take the 2005 Tulip Revolution in Kyrgyzstan as an example. Evolutionary theorists may argue that this revolution followed the natural evolution of Kyrgyz society. On the other hand, functionalist theorists may argue that there was disequilibrium in Kyrgyzstan at the time of the revolution. However, it would be beneficial to conceptualize social change the same way in order to be able to assess its impact on individuals. What is needed is a conceptualization of social change that can be interpreted in light of all the theories and processes that have been developed thus far. When an in-depth analysis of the literature is performed, the essential characteristics that define social change across theories may be ascertained. For example, one of the characteristics that was identified in conceptualizing DSC was the rapid pace of social change. The rapid vs. slow pace of social change is important, for instance, to distinguish a DSC from an incremental social change where transformations in the social structure take place without major disruptions. Whether one conceptualizes social change from a functionalist theory, a social identity theory, or a developmental theory perspective, most researchers from these distinctive fields point to the pace of change as one pivotal and essential element that characterizes DSC. Thus, when I base the typology of social change upon such characteristics, garnered from previous research in both sociology and psychology, an all-encompassing conceptualization of social change may be obtained, and later used to guide empirical research independently of the diverging theoretical perspectives.

My observations on the limitations of sociology and psychology should not detract from the insightful contributions these disciplines have made to our understanding of social change. Indeed, these social scientists have tapped into very important issues. For example, although collective action is not the only type of social change, the research on this topic has successfully identified factors that lead individuals and groups to be dissatisfied with their conditions and engage in collective action. However, as Sampson (1989) pointed out: “we have not gone far enough in connecting our theories of the person with social change, in particular, with major historic transformation in the social world” (p. 417). Since our contemporary social world is characterized by social change (Weinstein, 2010), like Sampson (1989), I argue that “a psychology for tomorrow is a psychology that begins actively to chart out a theory of the person that is no longer rooted in the liberal individualistic assumptions, but is reframed in terms more suitable to resolving the issues of a global era” (p. 431).

In sum, social change needs to be clearly examined because future research is limited without an all-inclusive typology of social change; one that can bridge the epistemological differences between theories from various fields of research and diverging theoretical perspectives. What is needed is a clear conceptualization of social change that considers, and includes, the different characteristics that compose DSC and that were suggested by researchers from all these diverging areas and theoretical orientations.

## Constructing a typology of social change: the characteristics of DSC

Two separate databases from sociology and psychology were targeted to collate relevant peer-reviewed publications: Sociology Abstracts and PsycInfo. Including the year 2016, a total of 5,676 abstracts were carefully analyzed (90% inter-judge reliability; Table 5). Two inclusion criteria were used to determine if a manuscript was relevant to our typology of social change. First, the selected abstract, and then the articles, needed to a) focus on social change by including a relevant original definition or providing an original perspective on the concept (originality), or b) focus on one's perspective of social change at either the individual or group level (perceptions).

**Table 5:** **Number of abstracts and articles that satisfied the specified inclusion criteria**.

	**PsycInfo**	**Sociology abstracts**	**Other**
Evaluated abstracts	2814	2862	
Accepted abstracts	250	178	
Missing articles	12	16	
Read and accepted articles	161	114	
Other articles and books			50
Total		325	

When reviewing the literature, I had one main goal: selecting and identifying the necessary characteristics of DSC that could either be present or not in other social contexts (i.e., stability, inertia, and incremental social change). Scientists refer to the characteristics in two different ways: (1) formally, when defining or describing DSC, incremental social change, stability, or inertia, and (2) informally, when introducing their research on social change^2^. I made sure that the included articles sufficiently addressed one or more of the four selected characteristics (i.e., rapid pace of change, rupture in social structure, rupture in normative structure, and threat to cultural identity, see Table 6). These four characteristics were chosen after a first reading of each of the articles (up to October 2013). They emerged most consistently and were singled out more often for their importance. From prior knowledge, I anticipated that “pace of change” and “social structure” would surface. The other two emerged naturally. From prior knowledge, I also expected the term “valence of change” (i.e., negative change) to emerge (e.g., Slone et al., 2002; de la Sablonnière and Tougas, 2008; de la Sablonnière et al., 2009c; Kim, 2008). However, that characteristic did not appear in a significant number of papers. The fact that some authors report “positive” change as having negative consequences (e.g., Prislin and Christensen, 2005; Bruscella, 2015) and “negative” change as having positive consequence (e.g., Yakushko, 2008; Abrams and Vasiljevic, 2014) may explain why the valence did not emerge as an important characteristic of DSC.

**Table 6:** **Characteristics of dramatic social change**.

**Characteristics**	**Definition**	**Occurrences**
1. The pace of change	The speed at which an event impacts a collectivity.	185
2. Rupture in the social structure	A break with the past so that even core aspects of society such as social institutions have to be reconstructed; a society undergoes a complete transformation.	196
3. Rupture in the normative structure	A break with the past in terms of the core behaviors of the group members that now have to be modified significantly in order to achieve collective goals.	195
4. Cultural identity threat	A serious threat to identification and to the clarity of the shared beliefs, values, attitudes, and behavioral scripts associated with one's group.	205

To conceptualize an event as DSC, all four characteristics must be present. For example, if an event is affecting only the normative structure in a gradual manner, it would not be possible to label that event as DSC. As for the other three social contexts (stability, inertia, and incremental social change), each has its own unique configuration of characteristics (see Figure 1)^3^.

### The pace of change

The first characteristic that emerged regards the *pace*, which could either be slow or rapid, and is defined as *the speed at which an event impacts a collectivity*. When defining social change, researchers from both sociology and psychology distinguish two types of social change based on the pace of change: incremental (e.g., first-order change, beta change, decline, gradual, small-scale) and dramatic (e.g., second-order, gamma, abrupt, collapse, large-scale).

Theories of social change have explicitly and/or implicitly acknowledged the pace of social change as a central determining factor toward its characterization. For example, in one of the earliest versions of their seminal book, Lenski and Lenski (1974) state: “The most striking feature of contemporary life is the revolutionary pace of social change. Never before have things changed so fast for so much of mankind” (Lenski and Lenski, 1974, p. 3, see also Fried, 1964; Rudel and Hooper, 2005). In their new edition entitled *Human Societies: An Introduction to Macrosociology*, Nolan and Lenski (2011) describe how slowly human evolution has progressed for thousands of years until about 100 years ago, when humans began to evolve at an accelerated pace. Similarly, Weinstein (2010) suggests that for the last few decades, there has been “rapid and accelerating rates of change in human relations, from the interpersonal to the international level” (p. xvii).

It is worthwhile to note that a few key authors refer to pace when distinguishing different types of social change. For example, in organizational psychology, Nadler and Tushman (1995) distinguish slow “incremental” change from fast “discontinuous” change, where the latter would be characterized as DSC in the typology of social change. According to these authors, incremental changes are intended to continually improve the fit among the components of an organization. These changes can either be small or large; nonetheless, there is a succession of manageable changes and adaptation processes. In contrast, discontinuous changes are often linked to major changes in the global scope of the industry and involve a complete break with the past as well as a major reconstruction of almost all elements of the organization. These changes are more traumatic, painful, and demanding as individuals are required to acquire a whole new set of behaviors and discard old patterns. These dramatic changes are not made to improve the fit, but to construct a new collectivity, be it a nation-state, institution or sub-group of the larger collectivity. Newman (2000) also distinguishes between first-order change and second-order change in the context of organizations. According to him, a first-order change, which is equivalent to incremental social change, “is most likely during times of relative environmental stability and is likely to take place over extended periods of time” (Newman, 2000, p.604). In other words, this type of change occurs slowly and allows the organization and its members to adapt to the changes gradually. However, a second-order change, or DSC, is radical, and transforms the core of the organization (Newman, 2000). In this case, the change is so sudden that it does not necessarily allow individuals to adapt to the process (Buchanan et al., 2005). Similarly, Rogers (2003) defines social change as abrupt and arises when the entire system is modified and jeopardized because changes are too fast for the system to adjust. In his book, Diamond (2005) contrasts “decline”—where minor ups and downs do not restructure the society—with “collapse”—an extreme form of several milder types of decline—which make it a DSC. An example of collapse is when most of the inhabitants of a population vanish as a result of ecological disasters, starvation, war, or disease. Examples of this are genocides such as Rwanda's which claimed around 800,000 lives, destroyed much of the country's infrastructure and displaced four million people (Des Forges, 1999; Zorbas, 2004; Pham et al., 2004; Staub et al., 2005; Schaal and Elbert, 2006; Prunier, 2010; Yanagizawa-Drott, 2014), the Armenian Massacres, which saw the systematic extermination of about 1.5 million minority Armenians in Turkey (Dadrian, 1989, 1998) or Cambodia's genocide, which involved the death of almost two million people through the Khmer Rouge's policies of relocation, mass executions, torture, forced labor, malnutrition, and disease (Hannum, 1989). All these events led to an inordinate number of deaths and population movements in a short, restricted period of time.

To be considered *dramatic*, a social change needs to be quick and must involve a “break with the past” (Nadler and Tushman, 1995; see also Armenakis et al., 1986). The example most often used in the literature is the breakdown of the communist system in Eastern Europe and the Soviet Union (e.g., Kollontai, 1999; Pinquart et al., 2009; Round and Williams, 2010; Walker and Stephenson, 2010; Chen, 2015). For example, when Pinquart et al. (2004, p. 341) introduced their research on social change, they made a distinction between “gradual” change, such as ideological change in many Western societies, and “abrupt social change,” which represents a form of social change that may be spurred by a sudden, dramatic transformation of economic, political, and social institutions.

### Rupture in the social structure

The second characteristic of DSC that emerges from my review regards a *rupture in the social structure* of a collectivity or a group. Social structure is a term that has several different uses in the sociological literature and this is, in part, because of the lack of agreement on how the term social structure should be defined (Porpora, 1989; López and Scott, 2000). One main dispute pits the dualism of “action” (or agency) vs. “structure” in mainstream sociological work (for a discussion see López and Scott, 2000). Consequently, many of the definitions describe behaviors rather than the role of social institutions (e.g., Cortina et al., 2012; Tanner and Jackson, 2012; Wilson, 2012). For example, Tanner and Jackson (2012) define social structure as “the formation of groups via connections among individuals” (p. 260), which focuses on meso-level interactions among individuals. Similarly, Macionis et al. (2008) define social structure as “any relatively stable pattern of social behavior” (p. 13).

The social structure being discussed in the present paper refers to macro-level elements of society such as institutions that facilitate and structure collective interactions, roles or behaviors. Thus, directly inspired from the most prominent definitions of social structure in the literature (Marx, 1859/1970; Giddens, 1979; Porpora, 1989; López and Scott, 2000; Stinchcombe, 2000), social structure is defined here as a *system of socio-economic stratification, social institutions, organizations, national policies and laws that help structure the norms, roles, behaviors, and values of community members*^4^.

In both sociology and psychology, a rupture in the social structure is at the heart of definitions of social change. For example, for Breakwell and Lyons (1996), changes involve the disintegration of previous national and international order and sets in motion a process of re-definition and re-evaluation of societal norms, belief systems, and power structures. While the communal sense of continuity and permanence is challenged, social change often represents a period of massive transformations in political, social, and economic structures (e.g., Goodwin, 1998; Kim and Ng, 2008; Chen, 2012). This conceptualization is similar to the definition inspired by sociologists and provided by Silbereisen and Tomasik (2010, p. 243) where “social change is understood as a more or less rapid and comprehensive change of societal structures and institutions, including changes to the economic, technological, and cultural frameworks of a society (Calhoun, 1992)” or to Kohn's definition of radical social change: “we refer not to the pace of change but to the nature of the change—the transformation of one political and economic system into a quite different system” (Kohn et al., 1997, p. 615).

When research focuses on collective action, social structure is placed at the root of their definition. For example, “Breakdown Theories” in sociology argue that social movements result from the disruption or breakdown of previously integrative social structures. This theory regards collective action as a form of social imbalance that results from the improper functioning of social institutions (Tilly et al., 1975). Macionis et al. (2008) also suggest that, “revolutionary social movements attempt to target the whole collectivity by radically changing social institutions” (p. 452). Put differently, for social movements and collective action to occur, social institutions—consequently, the social structure of society—needs to be altered. In other words, social change “is the sudden shifting of power from group to group” (Schrickel, 1945, p. 188). To many authors, DSC involves a rupture in the social structure (e.g., Prilleltensky, 1990) where people need to “negotiate their way through or around social structures” (May, 2011, p. 367).

### Rupture in the normative structure

The third characteristic of DSC that emerged from the literature is the rupture in the normative structure of society. While reading on the subject, I noticed an important distinction between social structure and normative structure. As mentioned in the previous section, that distinction pointed to a duality that is also observed by theorists in sociology who attempt to define social structure (e.g., Giddens, 1979; Mayhew, 1980; Porpora, 1989; López and Scott, 2000). Although both the social and normative structures refer to the functioning of a society, they each point to two different aspects of communities and groups. As discussed earlier, the social structure is associated with macro processes such as social institutions (e.g., Government), whereas the normative structure is related to micro processes as they principally refer to community members' habitual behaviors and norms.

Based on the work of Taylor and de la Sablonnière (2013, 2014), the normative structure is defined here as *the behaviors of most community members whose aim is achieving collective goals*. In other terms, when the normative structure is clear, people know what to do and when to engage in specific behaviors in order to meet the overarching goals of the collectivity. The definition of normative structure also takes its inspiration from an array of different domains in the scientific literature. Mainly, it comes from the definitions of social change that most often involve a change in behaviors and habits that are disrupted with the event of a dramatic and rapid social change. For example, Bishop (1998, p. 406) clearly states that social change in its transformational form refers to “the ability of a group to behave differently, even to creating brand-new elements, within the same social identity.” This definition concurs with definitions of many more authors, such as Delanty's (2012) concept of “normative culture” or May's (2011), where the mundane “ordinary” activities take a central place in social change.

Research and theories on social change have put normative structure as one of its central tenants. For example, Tomasik et al. (2010), argue that social change involves “changes of the macro-context that disturb habits, interrupt routines, or require novel behaviors relevant for a successful mastery” (p. 247). These authors also assert that when a gradual social change occurs, “old options of thinking and behaving are usually still available whereas abrupt social change is often associated with an immediate blocking of old options” (Pinquart and Silbereisen, 2004, p. 295). Therefore, in the latter case, it will be necessary to develop new ways of doing things.

Jerneić and Šverko (2001) argue that “major political and socioeconomic changes may strongly influence people's life role priorities, which are otherwise relatively stable behavioral dispositions” (p. 46). In fact, the normative structure of a society is comprised not only of norms and behaviors, but also of roles that people have in their everyday lives. When a DSC occurs, these normative elements of people's lives are all greatly affected to the point where they need to be redefined. Similarly, McDade and Worthman (2004) refer to “socialization ambiguity,” a state present in the context of DSC where “inconsistent messages or conflicting expectations regarding appropriate beliefs and social behavior during the course of socialization may be a substantial source of stress for the developing individual” (p. 52; see also Arnett, 1995; Tonkens, 2012).

This rupture in the normative structure of society is present not only when radical changes such as natural disasters occur, but also when social change is the result of collective actions within a society. Subašić et al. (2012) acknowledge that “what we do is evidently shaped by social norms, by institutional possibilities, and institutional constraints. But equally, we can act—act *together* that is—to alter norms, institutions, and even whole social systems” (p. 66). Therefore, when members of a society come together and engage in collective actions, an important aspect of society they aim to change deals with the norms and normative structure.

The importance of the normative component involved in DSC is in accordance with the Normative Theory of Social Change, developed by Taylor et al. (Taylor and de la Sablonnière, 2013, 2014; see also de la Sablonnière et al., 2009b). According to their theory, any group—whether it be at the collective, community or country level—functions along the basic *80-20 principle* in times of stability. According to this principle, most of the citizens in a functioning society (i.e., 80% of them) will exhibit normative behaviors that agree with the normative structure of the society in order to accomplish collective goals such as achieving a healthy society, and by extension, personal goals such as maintaining a healthy lifestyle. It is the 80% that provide social support, when necessary, to the 20% of citizens who do not function successfully in the society. In theory, as long as there is a decent majority of people who conform to the normative structure, a society should function relatively smoothly. Unfortunately, this is not always the case. Sometimes, when a society is confronted with DSC, its normative structure is ruptured which may lead to societal dysfunction or important disruptions in the “usual” behavior of group members. In such a situation, the amount of group members exhibiting behaviors that are in agreement with the collective goals of the group will be lower than usual. Therefore, it is possible that instead of having 80% of group members acting according to the normative rules of the society, only 30 or 40 % of individuals will follow these rules. In this case, it becomes very difficult for people to restore the functional equilibrium of the normative structure as only a few group members are in a position to provide the necessary social support for the entire society to function properly (Taylor and de la Sablonnière, 2014). What is suggested here is consistent with the work of Albert and Sabini (1974). These authors refer to the importance of a supportive environment, or social support, which has a sufficient presence in “slow change,” but not when the context is one of rapid change.

### Threat to cultural identity

The fourth characteristic of social change is threats to the cultural identity of a group. This characteristic is a difficult one to label since different authors use different terms to describe a threat to cultural identity (i.e., lack of clarity, identity conflict, identity crisis, lowered identification, identity confusion). As opposed to terms such as identity conflict, identity crisis, lack of identity clarity and identity change, “threat to cultural identity” was chosen for its capacity to suggest a potential modification in identity. To be considered DSC, the cultural identity in its current form must somehow be jeopardized, challenged, or lowered. Values and beliefs are, *per se*, questioned and the individual may sense a general lack of clarity and feel threatened to the core of his group identity, value system, or beliefs.

Many scientists have defined and researched collective and/or cultural identity. Recently, Ashmore et al. (2004) have defined collective identity as “first and foremost a statement about categorical membership. A collective identity is one that is shared with a group of others who have (or are believed to have) some characteristic(s) in common” (p. 81). This definition is similar to the one from Taylor (1997), in which cultural identity is referred to as the beliefs about shared rules and behaviors (Taylor, 1997, 2002; Usborne and de la Sablonnière, 2014).

When a social change occurs, it threatens the cultural identity of all community members. In the present paper, inspired from previous work on cultural identity, I define threat to cultural identity as *a serious threat to identification and to the clarity of the shared beliefs, values, attitudes, and behavioral scripts associated with one's group*. Throughout the literature I reviewed, cultural identity threat was manifested according to three main themes. The first theme that stood out is that threats to identity are associated with a loss of identity or an identity change (e.g., subtractive identification pattern; de la Sablonnière et al., 2016). Some authors directly mention the threat to cultural identity within the context of major social change (e.g., Vaughan, 1986; Smelser and Swedberg, 1994; Sztompka, 2000; Wyn and White, 2000; Van Binh, 2002; Terry and Jimmieson, 2003). For example, in his paper on how cultures change as a function of mass immigration Moghaddam (2012) argues that globalization results in sudden contact among different groups of people from different countries. This form of sudden contact has often resulted in the extinction of many cultures and languages such as Indigenous peoples around the world. Therefore, globalization makes people feel that their collective identity is threatened. Specifically, they experience a loss in many components of their cultural identity including their values and their language (see also Van Binh, 2002). The process described by Moghaddam is similar to the one proposed by Lapuz (1976) who argues that when social change occurs rapidly, people's beliefs and values are threatened since the old guidelines are no longer available. One consequence of this threat is that people become confused as values and beliefs contribute to the emotional security and psychological survival of individuals (Lapuz, 1976; Varnum, 2008). This is in agreement with Albert's (1977) proposition: “Rapid change constitutes a major threat to self-identity” (p. 499). Similarly, in their book entitled *Changing European Identities*, Breakwell and Lyons (1996) discuss the mechanisms associated with change in identities in the context of the development of the European Union and refer to a loss of national identity. This change in cultural identity is similar to what Wall and Louchakova (2002) describe as a “shift in the cultural collective consciousness” (p.253). This consists of a change in the American self and the emergence of new selves, more independent and alive in the context of change (see also Neves and Caetano, 2009; May, 2011).

The second theme is associated with the lack of identity clarity in the event of DSC. This lack of clarity is due to uncertainties or inconsistencies in the definition of one's identity. A clear cultural identity is defined as “the extent to which beliefs about one's group are clearly and confidently defined” (Usborne and Taylor, 2010, p. 883; see also Taylor, 2002). It has been theorized and demonstrated that an unclear cultural identity can result in lower self-esteem (Usborne and Taylor, 2010). Thus, if the entire collective is experiencing an unclear cultural identity, it may affect people's ability to function effectively in their society. Similarly, Macionis et al. (2008) refer to inconsistencies in the context of socialization in times of important change. People try to seek out new roles, try new “selves” (Macionis et al., 2008, p.461). They need to adapt to the inconsistent model their societies are projecting, which leads to “socialization ambiguity” (McDade and Worthman, 2004, p. 49). Because social change brings uncertainty in society, it can affect many aspects of individuals' lives such as family relations (Noak et al., 2001), and aspects associated with the self such as “emotions, values, perceptions, identity” (Wall and Louchakova, 2002, p. 266).

Finally, as a third theme, authors refer to conflicting identities within the context of dramatic contextual change. For example, Becker conducted a study to find out how rapid social change, such as introducing television in a community that had never owned televisions before, would impact body images of girls and women in that community (Becker, 2004). She found that television caused confusion and conflicts about ideal body images, and consequently “reshap[ed] [their] personal and cultural identities” (Becker, 2004, p. 551). In some cases, it even led to eating disorders (Becker, 2004), which has a direct link with the way people evaluate and perceive themselves. In other words, this DSC altered their identity. In fact, severe contextual changes can challenge the meaning of identity and threaten its existence (Ethier and Deaux, 1994; Macek et al., 2013). Similarly, Hoffman and Medlock-Klyukovski (2004) argue that contemporary organizations are “typically marked by conflicting interests and contradictory demands on individuals” (p. 389). This is similar to Chen (2012) who refers to the need for a transformation and the need to create new cultural norms and values when confronted to the context of social change (Chen, 2012).

## The typology of social change

In order to properly conceptualize DSC and other social contexts associated with the state of a collectivity, I suggest a typology of social change comprised of four different social contexts: “stability,” “inertia,” “incremental social change,” and “DSC” (see Table 1 for definitions). These social contexts are consistent with the theoretical stance of a large number of sociologists (e.g., Durkheim, 1893/1967, 1897/1967; Watzlawick et al., 1974; Rocher, 1992; Fukuyama, 1999; Rogers, 2003; May, 2011; Nolan and Lenski, 2011), psychologists (e.g., Katz, 1974; Moghaddam, 2002; Pinquart and Silbereisen, 2004; Goodwin, 2006; de la Sablonnière et al., 2009a) and scientists in the field of organizational behavior (e.g., Golembiewski et al., 1976; Tushman and Romanelli, 1985; Armenakis et al., 1986; Nadler and Tushman, 1995; Thompson and Hunt, 1996).

As many different concepts surround each of the four social contexts, it was necessary to choose a meaningful label for each. For “stability” and “inertia,” the choice was relatively easy because these two labels are commonly used and applied consistently. The term “status quo” was also considered rather than “stability” (e.g., Prilleltensky, 1990; Diekman and Goodfriend, 2007; Mucchi-Faina et al., 2010). However, because there could also be “status quo” in the context of inertia (e.g., Subašić et al., 2008), the term “stability” was preferred.

When it came to “incremental” and “dramatic” social change, the decision was more arduous as authors from different research fields use different labels. For example, instead of referring to “DSC,” Golembiewski et al. (1976) refers to “gamma changes”; Nadler and Tushman (1995), to “discontinuous change.” Others refer to “second-order change” (Watzlawick et al., 1974; Bartunek and Moch, 1987; Bate, 1994; Newman, 2000), to “abrupt” (e.g., Back, 1971; Pinquart and Silbereisen, 2004) or even to “rapid” change (e.g., Becker, 2004; McDade and Worthman, 2004). The term “dramatic” social change was chosen for its ability to clearly and distinctively define the situation confronting ordinary people. In a similar fashion, the term “incremental” social change was preferred over the labels: “first-order change,” “beta change,” and “continuous change.”

### Stability

When there is *stability*, the actual state of a society is maintained and the majority of group members are actively attempting to attain society's goals. As Weinstein (2010) describes it, it is a state in which “the established order appears to be operating effectively, and disturbing influences from within or from other societies are insignificant” (p. 9; see also Bess (2015) where no change is equated with stability). Indeed, none of the four characteristic of social change are present. For example, the social and normative structures fluctuate little, and changes do not affect what is defined as normal behavior in a community (Harmon et al., 2015). Indeed, personal change, such as bereavement or divorce, still occurs for some members of society. However, in the event of a personal change, the social or normative structures are not disrupted, mainly because the collective social support system remains functional and people can rely on that support in case they experience changes in their individual lives. This is also consistent with the findings of Albert and Sabini (1974) who argue that changes occurring in a supportive environment or in a peripheral element of society are perceived as less disruptive than those occurring in a non-supportive environment because the strain upon society is attenuated.

Consistent with previous research, *stability* can be defined as *a situation where an event, regardless of its pace, does not affect the equilibrium of a society's social and normative structures nor the cultural identity of group members. The event, may, however, impact an isolated number of individuals*. An example that might clarify this definition of stability is the event of an election. Although many people can get excited and seem to be affected by this event, an election does not necessarily bring about a rupture in a society, even if it involves a change of political party. The core elements of society remain stable and citizens resume their activities without feeling their lives have been overly disrupted by the election and its outcome. If, for instance, supporters of the defeated party feel sad and hopeless about the defeat, plenty of other citizens will be available to help them cope since most of them will not be affected by the change of government. However, in a different context, the event of an election may trigger DSC; for example, when it leads to a social revolution.

### Inertia

In contrast with stability, a context where there is *inertia* involves a situation that does affect a large number of people, if not most of the people composing a society. Inertia is defined as *a situation where an event, regardless of its pace, does not either reinstate the equilibrium of a society's social and normative structures or clarify the cultural identity of group members*.

In times of inertia, if a “positive” event occurs, there is no sustainability to maintain its positive impact. Here, the example of Belarus is used, a country where the population has been in a state of inertia since the fall of the Soviet Union. Lukashenko has been the president of the country since 1994. Under his autocratic rule, Belarus is known as the last dictatorship in Europe. Many Belarusians are longing for a more democratic and open society, yet the country remains in inertia. Buchanan et al. (2005) describe a situation of inertia as an “absence of appropriate activity, a lack of capability, a failure to pay attention to signals, and thus as an impediment rather than a desired condition” (p. 190). Inertia is seen as an undesirable situation where constructive change is not possible because the organization (or the group) does not have the capacity (e.g., lack of resources or will) to carry out the needed change. These authors also argue that when a change is implemented, its sustainability requires managers and staff (or community members) to share the same objectives. Uncertainty about the future must be minimal.

Accordingly, one can assume that the criteria underpinning sustainability in the event of a change are already absent in a society that has stagnated due to inertia. Therefore, inertia in a society such as Belarus constitutes a context where the population is uncertain about the future and does not share the same long-term goals as its government. There is a desire for positive social change, but the actual structure of the society makes it difficult for any change to be implemented and be sustained. Indeed, for a positive change to be maintained, it must have the support of individuals in power since they have the appropriate resources to address society's problems. Unsurprisingly, sustainability of such a change is threatened by an autocratic style of governing (Buchanan et al., 2005).

In sum, inertia differs from stability. In the case of inertia, most members of society desire a change from the actual state of their group, but are unable to properly sustain change due to a lack of collective social support and an unclear cultural identity. In contrast, in the case of “stability,” the society functions in an efficient manner when meeting the collective goals.

### Incremental social change

*Incremental social change* is defined as *a situation where a slow event leads to a gradual but profound societal transformation and slowly changes the social and/or the normative structure or changes/threatens the cultural identity of group members*. The *slow* pace is necessary for incremental social change to occur. Moreover, at least one of the other three characteristics needs to occur. In their recent paper, Abrams and Vasiljevic (2014) speak of “growth,” which could represent one form of incremental social change that involves “wider acceptance of shared values and tolerance of different values” and of “recession” where “disidentification” with current groups can occur (p. 328).

One of the most cited examples of incremental social change is technological innovation (e.g., Rieger, 2003; Weinstein, 2010; May, 2011; Hansen et al., 2012). Often, there is no social structural rupture associated with the wide use of technology and normative structure as well as social support remain intact. Given its incremental nature, this type of social change does not instantly produce conflict between old and new behaviors. For instance, when television was introduced, people bought it without knowing the consequences of the implementation of this new technology in their life (Becker, 2004; Macionis et al., 2008; Weinstein, 2010). Today, in retrospect, we know that buying a television set entailed a plethora of new behaviors that altered our society and our way of living. Indeed, some changes in society seem to be a “by-product of our pursuit of other goals and interests” (Subašić et al., 2012, p. 62). The long time span that is typical for incremental social change makes its outcomes unpredictable and unintentional. For instance, as Weinstein states (Weinstein, 2010), “It would be impossible to assess exactly what role electronic telecommunication has played in our global revolution, in part because its effects continue to reverberate and magnify as you read this” (p. 4).

The cell phone is a particularly good example of incremental social change. When it came onto the buyer's market, only a few exclusive people possessed one. However, over the years, it became increasingly normative to have a cell phone and, today, it is almost inconceivable not to have one. Furthermore, when cell phones were first marketed, they were used mainly for business rather than for social purposes, which is the current primary use (Aoki and Downes, 2003). In the same vein, other technological changes, such as the emergence of personal computers (Kiesler et al., 1984; Robinson et al., 1997), Internet (DiMaggio et al., 2001; Brignall III and Van Valey, 2005), and social media (Robinson et al., 1997; O'Keeffe and Clarke-Pearson, 2011; Oh et al., 2015) will, in the future, be recognized as key events in the historical transformation of social structures and social norms. Such technology does not represent a DSC, but a social change nonetheless as it has modified the way people interact with one another in an incremental manner. As the change occurs for a relatively long period of time, there is consistency in the pattern of change, which allows social structures to adapt and, thus, to remain intact (Nadler and Tushman, 1995). Individuals experiencing incremental social change are therefore able to adapt, given that the collective social support is not altered. For example, there is support for people that have yet to possess a cell phone; if they want to buy one, but do not understand how it functions, there are plenty of people that can help them adapt to this new technology. Even if technological change is conceptualized here as an incremental change, it is possible that technology is used to provoke a DSC, for example by instigating an important social revolution (Rodriguez, 2013).

Despite technology being the most adequate example, other incremental changes can be observed in other aspects of society such as in medicine. Indeed, advancement in medicine such as effective birth control (Goldin and Katz, 2002) was also the cause of a profound incremental social change. The example of contraception is crucial as the pill deeply affected gender roles in society by empowering women by giving them the capacity to control their sexuality. The pill had not only direct positive effects on women's career investments, but also on the opportunity of attending school longer. The pill forever changed women's involvement in our societies and the repercussions of this incremental social change still echo to us through struggles for gender equality, but also in the form of women actively involved in every level of the modern workplace, including higher managements and governmental position. In other words, the gradual nature of incremental social change makes it a profound change in society that neither disturbs the social structure nor the collective social support system.

### Dramatic social change

*DSC* has been defined as “profound societal transformations that produce a complete rupture in the equilibrium of social structures because their adaptive capacities are surpassed” (de la Sablonnière et al., 2009a, p. 325). Although this definition is based on previous sociological work (Parsons, 1964; Rocher, 1992), it is adapted here according to the four characteristic of DSC. Specifically, I suggest that DSC be defined as *a situation where a rapid event leads to a profound societal transformation and produces a rupture in the equilibrium of the social and normative structures and changes/threatens the cultural identity of group members*.

As with incremental change, DSC induces fundamental transformations in society. However, the shift occurs at a much more rapid pace, provoking a break with the past. Some authors have highlighted this sense of discontinuity by referring to DSC as the disintegration of a previous social order or as the break in a frame of reference (Golembiewski et al., 1976; Nadler and Tushman, 1995; Breakwell and Lyons, 1996). They also use terms such as the “construction of something new,” a “reconceptualization,” or a “re-definition.” Indeed, the breakdown of a social structure conveys the need for the reconstruction of core elements in a society. Accordingly, DSC can be conceptualized as a complete rupture in the social structure that marks the end of one period and the beginning of another one, or where a type of society is transformed into another (Tushman and Romanelli, 1985; Kohn et al., 2000; Weinstein, 2010). Other researchers, such as Rogers (2003), also see rapid social change as intertwined with the social structure. More specifically, Rogers (2003) states that rapid social change can threaten social structure by surpassing the adaptive capacities of individuals. Unsurprisingly, DSC is the most disruptive type of change not only for the social structure but also for the majority of society members experiencing it, i.e., the normative structure as well as cultural identities are challenged. As DSC entails a re-definition of values, norms and relations, individuals can no longer rely on their habits and routine; they need to learn new skills and new definitions and more challengingly, unlearn the old ways of doing things (Nadler and Tushman, 1995; Tomasik et al., 2010). Consequently, DSC is described as a painful and confusing experience for individuals (Hinkle, 1952; Lapuz, 1976; Nadler and Tushman, 1995; Kohn et al., 2000; Wall and Louchakova, 2002; Rioufol, 2004; Hegmon et al., 2008).

A good example of DSC is the breakdown of the Soviet Union. If I return to Zoia's example, it is clear that all the people in Kyrgyzstan and in the Former Soviet Union were affected by the breakdown of the Soviet Union. Zoia is not the only one who lost all her savings: the vast majority of people lost their savings within a matter of days. In terms of social support, whom could she have relied on if all of her friends were also in the same situation? Regarding to the fall of the former Soviet Union, Goodwin (2006) argues that older people were inclined to receive less social support in part because the majority of the population, including family members, were struggling with several jobs just to provide themselves with basic needs. Furthermore, elderly citizens could not even rely on formal social services because the collapse of the former Soviet Union caused a decline in formal state support, which left them no time to rebuild their retirement income. This illustrates the rupture in the structure of society that can be found when a DSC occurs as well as the effect on the majority of ordinary group members who cannot rely on collective social support.

## Coming full circle: theoretical implications

Heraclitus, an ancient Greek philosopher, is credited for saying that “the only thing constant is change.” Gradually or within an instant, civilizations, societies, communities or organizations that often seem immutable face multiple DSCs. Social scientists agree that social changes are not only intensifying but also defining today's world. In fact, Weinstein (2010) has underscored that “rapid change, both peaceful and violent, is a fact of life that virtually everyone on Earth today has come to expect, if not unconditionally accept” (p. 3).

For the present paper, my aim was to initiate a conversation about the psychology of social change. Thus, I briefly reviewed the major perspectives of social change in both sociology and psychology. Research conducted in both fields and their subfields have remained in distinct silos with no effort made toward aggregating their findings. This has unfortunately resulted in the absence of an encompassing approach in the current literature of social change: social change has never been integrated into a single perspective that would define or contextualize DSC within the spectrum of different social contexts. More importantly, social change has not been conceptualized so that micro processes, macro processes, and the important relations between them are addressed. As a result, the *typology of social change* introduces different social contexts (e.g., stability) that can serve as a basis of comparison for DSC. Based on my review of the literature, I suggest four necessary characteristics of DSC (Table 6).

The present paper then offers a first *step* toward unifying the variety of theories of social change which are currently isolated from each other. Indeed, our approach aims at addressing the challenge raised by Sun and Ryder (2016) concerning our need for “a more nuanced understanding of rapid sociocultural change combined with sophisticated research methods designed to address change in a multilevel way” (p. 9). The typology of social change I am suggesting is an emerging concept; thus, I invite debate with the hope that the views presented here will stimulate others to contribute to a needed understanding of DSC within an individual perspective. More importantly, based on such a typology of social change, theoretical models could be suggested as they might offer a guide to understanding the consequences of social change. For instance, such theoretical models could answer these three questions: Are the different social contexts associated with one-another? What makes a society move from one social context to another (e.g., from stability to DSC)? What is the role of the different characteristics of DSC? So far, answers to the three questions raised above were left lingering and the different characteristics of DSC were not arranged in a sequential way nor were they identified as key movers of one state of society to another. In Figure 1, I offer a theoretical model that integrates the social contexts and the characteristics of DSC as a first step toward a psychology of social change.

As seen in Figure 1, neither a slow nor a fast pace event will influence the status quo in both stability and inertia. There will therefore be no break with the past and so no rupture in the social and normative structures. Thus, in these two social contexts, if an event were to occur rapidly, the current situation of a group or society would remain unaffected by it; that is why pace is not the only characteristic important to define DSC. For example, if a plane crashes, which is a rapidly occurring dramatic event, it does not necessarily affect an entire community. Also, in a state of stability, when a fast—or slow—event takes place, because the normative and the social structures are unaffected, there is no direct threat to the group's cultural identity. Similarly, when an event occurs in a state of inertia, there is no additional threat to the society's cultural identity, because the normative and social structure are unaffected.

In contrast, in a state of incremental social change, slow-occurring events, if profound enough, will gradually change the social and normative structures, as well as threaten or change cultural identity. For a DSC to occur, a fast event needs to take place. If that event has enough impact—therefore not in a state of stability or inertia—, it will rupture the social structure and the normative structures. As shown by many different DSC contexts, there are three possible scenarios when it comes to the rupture of these two structures: (1) the social structure ruptures first, which later leads to the rupture of the normative structure (e.g., Zhang and Hwang, 2007), (2) the normative structure ruptures first, which later leads to the rupture of the social structure (e.g., Centola and Baronchelli, 2015), or (3) both the social and normative structures rupture simultaneously and influence each other.

An example of the first scenario would be the latest presidential elections in the United States. The recent proclamation of Donald Trump as president carries the potential for political transformations as well as changes in the United States' economic structure (rupture to social structure). The leadership of Trump's administration can carry major structural change that would then lead to a rupture of the normative structure. At this point, there are indications that this new governance (social structure) may very well affect the normative structure. Some members of the population have become more “open” to expressing their reluctance to have more immigrants come to the USA, which could eventually lead to a rupture in normative structure where different ethnic groups overtly fight each other within America. A second example was the loss of the French Canadians to the English Canadians at the Battle of the Plains of Abraham in 1759. This battle was a pivotal moment in the 7 Years' War and gave power to the British troops (Veyssière, 2013). The result of the battle culminated in the French losing most of their economical structural powers to the English and the start of a decline of education. Consequently, the French mentality and behaviors were modified. The norms had to be adapted to new rules and to the loss of economic power (Veyssière, 2013).

The normative structure can rupture before the social structure in situations such as the African-American Civil Rights Movement in the United-States, the Fall of Apartheid in South Africa, or the Quiet Revolution in Québec. If in the past African-Americans were afflicted by a sense of resignation, leaders such as Martin Luther King Jr. and Rosa Parks gave them the will they needed to fight for a better future for themselves. This rupture in the normative structure led to the African-American Civil Rights Movement which, in turn, brought about changes to the social structure (e.g., School desegregation). This movement against racial inequality, segregation and discrimination instigated the Civil Rights Act of 1964, which banned any type of segregation based on race, color, religion or sex, as well as other changes in federal legislation.

The breakdown of the Soviet Union is an example that can be used to illustrate a simultaneous rupture of the social and normative structures. This event caused major transformations in the economic, political, and social structures (rupture to social structure). Simultaneously, a large proportion of the population found themselves in a great economic crisis, which led to disruptions in their usual behaviors and habits, such as working multiple jobs instead of just one (rupture of normative structures).

When the normative and the social structures are ruptured (regardless of the order in which this occurs), cultural identity will be threatened. There will be a global sense of confusion, ambiguity, and lack of clarity that might motivate individual group members to change their identification with their group.

Depending on society's and the individual's abilities to cope, there are two possible outcomes: stability or inertia. If the society in which DSC has taken place is able to develop coping and adaptation mechanisms—both at the individual and societal levels—stability might be restored. Stability would then be achieved when the social and normative structures however different are brought back to functionality and when cultural identity is clear and no longer under threat. In contrast, if the society and individuals are not able to develop coping mechanisms, society might enter a state of inertia. In inertia, even though a society in a state of inertia is no longer going through major social changes, the need or desire for change still lingers (Sloutsky and Searle-White, 1993). This can be due to a DSC that did not, in the end, really change the way a collectivity is ruled or how its citizens are treated (Moghaddam and Crystal, 1997; Moghaddam and Lvina, 2002).

## Consequences of DSC

Knowing about the range of different social contexts such as stability, inertia, incremental change, and DSC as well as the specific characteristics of DSC, has the potential to guide researchers in terms of assessing DSC and its impact on the psychological well-being of ordinary group members. Specifically, after establishing a clear typology of social change, including potential theoretical models, it is now possible to move on to the *second step* of the psychology of social change. In this second step, we need to address whether and how different coping mechanisms determine (mediate, moderate) the influence of DSC on psychological well-being. This question goes hand in hand with the work of Norris et al. (2002) who reviewed 160 studies involving natural disasters, mass violence, and technological disasters. They concluded from more than 60,000 participants that such events have negative repercussions on participants' lives. In most of the research they report, social support, economic status, and age were the identified factors that may be associated with a better adaptation to social change. Although diverse factors were suggested, the research they reported was “atheoretical and little of it is programmatic” (Norris et al., 2002, p. 249). In accordance with Norris et al. (2002), I argue that the mediators or moderators involved in adaptation mechanisms should become the focus of future studies. The four characteristics I have identified have the potential to become pivotal in meeting this objective. In sum, the link between social change and well-being is still unclear (e.g., Liu et al., 2014; Sun and Ryder, 2016). Such an investigation could eventually guide us in designing concrete interventions to help people adapt to the challenges of DSC (Rogers, 2003; Vago, 2004).

The concept of resilience emerges from the literature as potentially useful for understanding people's coping mechanisms. Resilience is defined as the act of bouncing back in the face of adversity (Bonanno, 2004). For the specific example of DSC, resilient individuals would be those who have been able to maintain their normal functioning and adapt themselves to adverse situations (Masten, 2001; Curtis and Cicchetti, 2003; Luthar, 2003; Masten and Powell, 2003). Research has shown that a significant number of people are able to adapt to challenging personal situations (e.g., Bonanno, 2004). However, resilience has mostly been studied within the context of personal changes such as the death of a loved one or a personal trauma (Bonanno, 2004). Similar to a personal change, this variation in reactions may be due to individual differences in resilience. This highlights the need to consider this variable within the psychology of social change. More concretely, the literature on resilience may prove to be important when linking people's perceptions of the characteristic of DSC to the various paths of recovery (e.g., resilience, recovery, chronic distress, and delayed reactions; Bonanno, 2004).

While most research on resilience focuses on “personal events,” there is, however, another type of resilience known as “collective resilience” or “community resilience” (e.g., Landau and Saul, 2004; Kirmayer et al., 2011) which may be more relevant in the context of DSC as the concept hints that the majority of society is affected by the change. To illustrate collective resilience, let us consider the case where the normative structure of a society is dissolved and its cultural identity is threatened. Individuals in this situation would no longer have guidelines and values to individually cope with DSC. Moreover, every individual affected by the change would be in the same negative situation. Consequently, individuals might need to find ways to collectively adapt to the transformations. The processes associated with resilience may thus differ in situations of personal vs. social change. I therefore believe it is important to explore whether the adaptation mechanisms are the same in a context of DSC where social support is not readily available.

## Conducting research on social change

In order to speak of a real psychology of social change, we must be able to actually study social change and its consequences. The use of a mix of methodologies that would include large correlational or longitudinal surveys conducted in the field as well as laboratory experiments (de la Sablonnière et al., 2013; see also Liu and Bernardo, 2014; Sun and Ryder, 2016) might prove to be the only way to truly study social change and its consequences. On the one hand, correlational designs conducted in the field are necessary to capture people's firsthand experience with DSC. They are however limited by their design that prevents claims of causality. They are also known to be demanding in terms of both human and financial resources, and may well be dangerous at times for researchers. Moreover, they require an intimate knowledge of the culture such as the language as well as contacts within the community to facilitate the research and collaboration process.

On the other hand, laboratory experiments are necessary to establish the controlled conditions needed to understand associations between the characteristics of social change and the consequences. Laboratory experiments, however, are difficult to design, because it is a challenge to reproduce the actual characteristics of social change in the laboratory which limits their ecological validity (de la Sablonnière et al., 2013). Indeed, social change typically entails various elements such as historical processes, a collective perspective, and associated cultural elements (Moghaddam and Crystal, 1997) which must be taken into consideration in order to replicate their impact in an artificial setting. For example, the impact of the Tohoku tsunami in Japan or the Syrian conflict cannot be recreated in their entirety in a laboratory; nor can all the characteristic of social change be taken into consideration in a laboratory study designed to assess the impact(s) of social change. However, if an array of studies using different characteristic of DSC were to be conducted (or a combination of multiple characteristic), the convergence of the results would make us able to better understand and thereby predict the impact of DSC on individuals and communities. At the very least in a laboratory, researchers can expose participants to imagined changes through a scenario or a video that would include, in the experimental condition, one or more of the four characteristics of DSC (Pelletier-Dumas et al., submitted). If the scientific community accepts that experimental studies will not exactly mirror DSC, but instead test some of the characteristics in a large number of experiments, there is potential for laboratory experiments to bring an important contribution that would eventually allow a generalization to the real world (for examples see Betsch et al., 2015; Caldwell et al., 2016; Pelletier-Dumas et al., submitted).

The difficulties of conducting research on social change are, however, amplified by the challenge of obtaining ethical consent in a manner that allows for timely research. In terms of experimental manipulations of DSC, obtaining the ethical board's consent can be tedious. Indeed, according to some authors (Kelman, 1967; Bok, 1999; Clarke, 1999; Herrera, 1999; Pittenger, 2002) deceiving participants is difficult to justify ethically. This objection on the use of deception can undermine any attempt to seriously study DSC, as deception can be a valuable methodological asset (Bortolotti and Mameli, 2006), especially with such an elusive subject. Furthermore, research on new grounds require new techniques and methods on which ethicists can put limits, to ensure that they do not cause harm to participants (Root Wolpe, 2006). As with any new technology, methods focused on inducing dramatic-like changes can be perceived as having unsuspected risks.

## Conclusion

In order to truly understand the interplay between individuals and their context, social psychological theories must take into account that we live in a constantly changing world. Unfortunately, although social psychology was rooted in understanding social change, most modern psychological theories refrain from addressing a “true” psychology of social change and prefer relegating social change to the field of sociology.

Through increasing the focus on social change, we could combine, on the one hand, sociology's emphasis on the importance of social change with, on the other hand, psychology's emphasis on the importance of complex individual processes. As a result, my theoretical proposal aims at bringing together sociology, where social change is central, and psychology, where rigorous scientific methods allow us to study the psychological processes of individuals living in changing social contexts.

In general, more research on the concept of social change is needed so that we can help predict, prevent, and minimize the negative impact of social change. If psychologists and sociologists work together to move toward developing a psychology of social change, perhaps we could come to better understand and help people, like Zoia, who lost almost everything they had, consequently improving the quality of millions of lives experiencing DSC.

## Author contributions

RdlS thought and developed the ideas, as well as wrote the article as sole author. Research assistants were paid to find and read the abstracts of all articles reviewed in this manuscript.

## Funding

This research was founded by a grant from the Social Sciences and Humanities research Council of Canada (SSHRC) and by a grant from the Fonds de recherche du Québec – Société et culture (FRQSC).

### Conflict of interest statement

The author declares that the research was conducted in the absence of any commercial or financial relationships that could be construed as a potential conflict of interest.

## Footnotes

^1^Key sociology readings, such as *Human Societies: An Introduction to Macrosociology* (Nolan and Lenski, 2011), *The Sociology of Social Change* (Sztompka, 1993), and *Social Change* (Weinstein, 2010), offer an in-depth description of these theories that were beyond the scope of the present review.

^2^For space limitation, all methodological details and steps I encountered are not discussed here, but are available upon request to the author.

^3^The term “social change” must be distinguished from the term “event.” An event may or may not be considered as social change. An event has the potential to bring social change (Sewell, 1996), be it incremental or dramatic. However, an event is not always tied to social change as it may represent a form of “happening” that does not influence the course of history (Nisbet, 1972). In sum, an event is an intrusion or “disturbances, however mild, of the normal” (p. 26). In contrast to social change, with the event, the disruption of the normal might only be temporary and not significant in time.

^4^Defining social structure represents a challenge that goes beyond the scope of the present paper. From my understanding of the literature, there are as many conceptions of social structure as there are scientists working on that concept. The most important issue that demonstrates how hard it is to define social structure is the fact that one of the most prominent sociologists, Giddens (1979), refers to a “duality of structure” when defining social structure (structure vs. agency). On the one hand, social structure represents institutions or more specifically “collective rules and resources that structure behavior” (Porpora, 1989, p. 195). Here, scientists refer to “groups, institutions, laws, population characteristics, and set of social relations that form the environment of the organization” (Stinchcombe, 2000, p. 142), or to “Lawlike regularities that govern the behavior of social facts” (Porpora, 1989, p. 195). On the other hand, social structure represents “the underlying regularities or patterns in how people behave and in their relationships with one another” (i.e., agency; Giddens et al., 2011, p. 3). Here, the definitions often described normative behaviors or the roles of individuals rather than the role played by social institutions (e.g., Cortina et al., 2012; Homans, 1951; Mayhew, 1980; Tanner and Jackson, 2012; Wilson, 2012). This duality lunched a debate in sociology that was reflected not only in Gidden's work but also in others sociologists that have devoted their writings to defining social structure (e.g., Parsons, 1964; Mayhew, 1980). For example, Porpora (1989) reports four principal ways of conceptualizing social structure that reflect either of these conceptions. More recently, expending on the work of Bourdieu (1975) and of Goffman (1983), López and Scott (2000) proposed that there is another aspect of social structure that must also be considered in addition to the institutional and relational structures: the embodied structure described as the “habits and skills that are inscribed in human bodies and minds” (p. 4). To add to that complexity, some researchers (e.g., Bronfenbrenner, 1979, 1994; for other “system views” see for example Marx, 1859/1970; Habermas, 1987) describe the possible “systems” that are, like Russian dolls, embedded in each other. These systems include the ecological environments “conceived as a set of nested structures” (Bronfenbrenner, 1994, p. 39): the microsystems, the mesosystems, the exosystems, the macrosystems, and the chronosystems. This “ecological model” illustrates the complexity of social structure as a sociological term. Because of the lack of clarity, or maybe because the definition of social structure points to different aspects of the social structure, scientists often avoid defining social structure in their papers, and thereby contribute to the general confusion. Not that the other aspects or levels of social structure are not important (e.g., meso, micro), but the social structure being discussed in the present paper refers exclusively to macro-level elements of society such as institutions and other environmental factors that help facilitate and structure collective interactions, norms, roles, and behaviors.
